# Increased hippocampal volume and gene expression following cognitive behavioral therapy in PTSD

**DOI:** 10.3389/fnhum.2013.00747

**Published:** 2013-11-07

**Authors:** Ahmed A. Moustafa

**Affiliations:** Marcs Institute for Brain and Behaviour, School of Social Sciences and Psychology, University of Western SydneySydney, NSW, Australia

**Keywords:** PTSD, hippocampus, amygdala, neurogenetics, cognitive behavioral therapy, computational modeling

In a recent study in *Biological Psychiatry*, Levy-Gigi et al. found that a 12 weekly 90-mins cognitive behavioral therapy (CBT) treatment in individuals with posttraumatic stress disorder (PTSD) is associated with an increase in hippocampal volume and expression of glucocorticoid receptor genes, known as FKBP5 (Levy-Gigi et al., 2013). This is one of the few studies that have investigated the effects of CBT on changes in brain volumes as well as gene expression in individuals with PTSD. The Levy-Gigi et al. study complements prior studies showing that antidepressant treatment is also associated with increased hippocampal volume in psychiatric patients, including PTSD and depression (Vermetten et al., 2003). Despite the positive effect of psychopharmacological and behavioral therapy in PTSD, it is not known whether CBT and psychopharmacological treatments are associated with dissociable effects on the brain and behavior.

Interestingly, Levy-Gigi et al. found that CBT ameliorates all aspects of PTSD, including avoidance, re-experiencing, and hyperarousal symptoms. However, it is not clear from the Levy-Gigi et al. study whether changes to the hippocampal volume are associated with amelioration to which PTSD symptoms. Prior studies show that there is a trend for a negative correlation between higher re-experiencing symptoms and hippocampal volume (Shucard et al., 2012), perhaps suggesting that re-experiencing symptoms are more ameliorated than other PTSD symptoms following CBT treatment.

At the neural level, several studies show that PTSD is associated with abnormalities to various brain areas, including reduced activity and volume of the hippocampus (Gilbertson et al., 2002; Smith, 2005), reduced activity of the ventromedial prefrontal cortex (Shin et al., 2005; Phan et al., 2006), increased activity of the dorsal anterior cingulate cortex (Shin et al., 2011), and increased activity of the amygdala (Armony et al., 2005; Shin et al., 2005). However, why would CBT lead to changes to the volume of the hippocampus but not the amygdala or other cortical structures, as found in the Levy-Gigi et al. study? Prior studies have shown the hippocampal volume is a risk factor for the development of PTSD (Gilbertson et al., 2002), while changes to amygdala, the ventromedial prefrontal cortex, and dorsal anterior cingulate cortex are acquired following trauma exposure. This perhaps suggests that changes to hippocampal volume following CBT can help protect against future occurrences of PTSD symptoms.

How can hippocampal dysfunction be related to PTSD symptoms? As noted by Levy-Gigi et al., the ventral hippocampus-amygdala pathway has been shown to be related to increased stress and anxiety (Fanselow and Dong, 2010). Further, many animal and human studies show that the hippocampus is involved in contextual fear responses (Anagnostaras et al., 1999; Corcoran and Maren, 2001; Ji and Maren, 2007; Acheson et al., 2012), such that hippocampal damage leads to increased fear response regardless of the context. fMRI studies have also reported greater hippocampus activation during contextual than cue conditioning (Marschner et al., 2008). In a prior computational model, Moustafa et al. (2013) show that the hippocampal region processes contextual information and sends representations of context to the basolateral amygdala and ventromedial prefrontal cortex for fear acquisition and extinction learning [for a review, see Rudy et al. (2004); Goosens (2011)], which help decrease and increase fear responses within that context. It is also not clear in the Levi-Gigi et al. study how changes to the hippocampal volume ameliorate PTSD symptoms. Moustafa et al. (2013) suggest that hippocampal projections to the ventromedial prefrontal cortex and/or basolateral amygdala could be enhanced by CBT treatment, which then lead to a decrease in fear responses when faced with a reminder of the trauma. This hypothesis can be confirmed or disconfirmed using diffusion tensor imaging in both individuals and animal models of PTSD [see for example Ding et al. (2013)].

The Levy-Gigi et al. study also showed that CBT alters the expression of genes, including the FKBP5 gene. FKBP5 regulates glucocorticoid receptor sensitivity, and reduce the efficacy of cortisol in the brain (Mahon et al., 2013). Polymorphisms in the FKBP5 gene relate to differences in glucocorticoid receptor sensitivity and stress hormone system regulation (Menke et al., 2013) as well as recovery from stress disorders (Ising et al., 2008) and rapid response to antidepressant treatment (Binder et al., 2004). As noted by Levy-Gigi et al., individual differences in the FKBP5 gene are associated with anxiety disorders, including PTSD (Xie et al., 2010; Boscarino et al., 2012).

Along these lines, Felmingham et al. (2013) found that individual differences in the brain-derived neurotrophic factor (BDNF) gene predicts the efficacy of exposure therapy in individuals with PTSD. It remains to be shown whether BDNF gene expression also undergoes changes following either CBT or exposure therapy, and whether exposure therapy affects the hippocampal volume.

Future computational modeling work is needed to explain how changes to the hippocampal volume and gene expression can ameliorate PTSD symptoms [see for example Krasne et al. (2011); Li et al. (2011); Moustafa et al. (2013)]. These computational models should also tie together brain volume and gene expression data in one framework to explain how clinical treatments can ameliorate symptoms in PTSD.

In summary, Levy-Gigi et al. (2013) have extended prior studies and shown that the hippocampus is a key brain structure that benefits from CBT. Future work should focus on network changes of hippocampus projections to the amygdala and ventromedial prefrontal cortex during behavioral and psychopharmacological treatments in PTSD.
